# Ethical problems in an era where disasters have become a part of daily life: A qualitative study of healthcare workers in Turkey

**DOI:** 10.1371/journal.pone.0174162

**Published:** 2017-03-20

**Authors:** M. Murat Civaner, Kevser Vatansever, Kayihan Pala

**Affiliations:** 1 Department of Medical Ethics, Uludag University School of Medicine, Bursa, Turkey; 2 Department of Medical Education, Ege University School of Medicine, Izmir, Turkey; 3 Department of Public Health, Uludag University School of Medicine, Bursa, Turkey; Centre for Injury Prevention and Research, Bangladesh (CIPRB) & Örebro University, Sweden, BANGLADESH

## Abstract

**Background:**

Natural disasters, armed conflict, migration, and epidemics today occur more frequently, causing more death, displacement of people and economic loss. Their burden on health systems and healthcare workers (HCWs) is getting heavier accordingly. The ethical problems that arise in disaster settings may be different than the ones in daily practice, and can cause preventable harm or the violation of basic human rights. Understanding the types and the determinants of ethical challenges is crucial in order to find the most benevolent action while respecting the dignity of those affected people. Considering the limited scope of studies on ethical challenges within disaster settings, we set upon conducting a qualitative study among local HCWs.

**Methods:**

Our study was conducted in six cities of Turkey, a country where disasters are frequent, including armed conflict, terrorist attacks and a massive influx of refugees. In-depth interviews were carried out with a total of 31 HCWs working with various backgrounds and experience. Data analysis was done concurrently with ongoing interviews.

**Results:**

Several fundamental elements currently hinder ethics in relief work. Attitudes of public authorities, politicians and relief organizations, the mismanagement of impromptu humanitarian action and relief and the media's mindset create ethical problems on the macro-level such as discrimination, unjust resource allocation and violation of personal rights, and can also directly cause or facilitate the emergence of problems on the micro-level. An important component which prevents humanitarian action towards victims is insufficient competence. The duty to care during epidemics and armed conflicts becomes controversial. Many participants defend a paternalistic approach related to autonomy. Confidentiality and privacy are either neglected or cannot be secured.

**Conclusion:**

Intervention in factors on the macro-level could have a significant effect in problem prevention. Improving guidelines and professional codes as well as educating HCWs are also areas for improvement. Also, ethical questions exposed within this study should be deliberated and actualized with universal consensus in order to guide HCWs and increase humane attitudes.

## Introduction

The classical definition of the term "disaster", which provides a clear distinction from "emergency" is “A situation or event which overwhelms local capacity, necessitating a request on the national or international level for external assistance” [1]. This imbalance hampers healthcare systems due to the increased needs of population, direct damage on infrastructure and loss of healthcare workers (HCWs). HCWs work in conditions different than their daily routine, as there are a number of stressors such as heavy workloads, limited resources, security concerns regarding themselves, relatives, and patients, absence of firm guidance in international law and health policy and the diversity of cultural backgrounds and language barriers [2–4]. A dramatic environment which demands urgent and vital action might pose different kinds of value problems while making life and death decisions by triaging patients, coping with the problems related to relief, or carrying out research within the affected population.

Since modern disasters, natural or human-made occur more frequently, causing more deaths, affecting more people and increasing economic loss [5–7], understanding the nature of the ethical challenges specific to disaster settings is becoming more important to prevent ethical conflicts or to be helpful to all parties to find the least negative option for action under severe conditions. In a study examining how HCWs experience ethics during the course of humanitarian assistance, Hunt described the core themes as “tension between respecting local customs and imposing values; obstacles to providing adequate care; differing understandings of health, illness and death; questions of identity for health workers relating to being a moral person; and issues of trust and distrust between humanitarian workers and the local community” [3]. Schwartz et al defined “(a) resource scarcity and the need to allocate them, (b) historical, political, social and commercial structures, (c) aid agency policies and agendas, and (d) perceived norms around health professionals’ roles and interactions” as the sources of ethical challenges [8]. Hunt, in another study, found that the resources for ethics deliberation and reflection include “opportunities for discussion, accessing and understanding local perspectives, access to outside perspectives, attitudes, such as humility, open-mindedness, and reflexivity, and development of good moral “reflexes.” [9]

However studies in the literature which aim to reveal the types, nature, and the context of ethical challenges HCWs face in disaster settings are very limited. Current studies were carried out with expatriate humanitarian workers [2, 3, 8]. However, it is emphasized that defining ethical practice in humanitarian healthcare is problematic if it does not consider values and perceptions of national staff [10], so understanding the experiences and perceptions of local HCWs is important “to gain fuller understanding of the elements involved in ethical challenges in humanitarian aid” [8], and “to increase trust, respect, and dialogue with humanitarian workers" [10]. Also, further research is required covering a more representative sampling of health professionals and disaster types.

Considering this gap in the literature, we carried out a qualitative study among local HCWs experienced in disaster settings, and focused on the types and nature of the ethical problems they face in disaster settings along with the factors causing or facilitating the emergence of them. We believe gaining an understanding of the determinants of ethical challenges could be helpful toward prevention and preparedness, and could provide significant tools for developing guidelines and educational programs. In addition, a study to be conducted on the national level might provide insight for foreign relief workers and organizations about how their activities are perceived by local HCWs and communities.

## Materials and methods

### Study design

Considering the necessities of the aims described above, a qualitative approach based on the Grounded Theory methodology was chosen chiefly to capture the contextual details. The study was carried out in Turkey, a country which lies between Europe and Asia with a population of 78 million. Turkey is frequently hit by almost all types of natural, technological and complex disasters. Since the Marmara earthquake in 1999, which was a major disaster in its history killing 18,000 people, 157 disasters have occurred in Turkey affecting 2.5 million people [11]. Turkey has often faced massive waves of refugees, especially in the last three decades; more than 300,000 people from Bulgaria in 1989, thousands of people from Iraq during the Gulf War in 1991, 2,500,000 Syrians and over 250,000 from other nationalities who have come to Turkey as refugees since 2011 [12–14].

The method of measurement used in this qualitative study was in-depth interviews using a semi-structured form consisting of three main topics: a) participants' experience in disasters, b) training related to disasters and c) ethical problems encountered during disasters. Interviews were carried out by the first two authors, MMC and KV, in six cities of Turkey; Istanbul, Ankara, Izmir, Bursa, Adana, and Antakya. The first five are the largest cities and have also been exposed to major disasters and have most of the experienced relief workers working in various institutions. Antakya was chosen because it is one of the cities most affected by the migration crisis and war in Syria, and its hospitals have had to face the heavy burden of treating wounded members of armed groups.

A purposeful sampling based on a maximum variation method was chosen in order to reach HCWs with diverse professional backgrounds and experienced in various types of disasters. Another criterion for determining the number of participants was saturation of information. Considering these criteria, 33 HCWs were determined by a snowball sampling technique starting from the disaster response team members of the Turkish Medical Association and Ministry of Health, two of whom declined to participate to the study considering the political dimension of the questions. Therefore 31 HCWs were interviewed between March-May 2014, including physicians whose specialties were general practice, public health, pediatrics, pediatric surgery, cardiovascular surgery, general surgery, psychiatry, anesthesiology and reanimation and medical education. Also a nurse, a psychologist and two medical students were interviewed.

Disasters settings that the participants involved as HCWs were;

- Earthquakes *(Erzurum-1983*, *Adana-1998*, *Marmara-1999*, *Düzce-1999*, *Afyon-2002*, *Bingöl-2003*, *Simav-2011*, *Van-2011)*
- Floods *(Izmir-1995*, *Bartın-1998*, *Istanbul-2010)*
- Avalanche (*Erzurum-1985*, *Siirt-1992)*
- Chemical explosions *(Izmir-1995)*
- Mine accident *(Soma–2014)*
- Explosions *(Kırıkkale-1997*, *Reyhanlı-2013)*
- Armed conflict *(Antakya- since 2011)*
- Refugee camp *(Kırklareli-1999)*
- Outbreak *(Crimean–Congo hemorrhagic fever–2011)*
- Mass protests *(All over Turkey*, *esp*. *Istanbul*, *Ankara and Izmir*, *2013)*
- International *(Pakistan earthquake-2006*, *Somali-2011*, *Sudan-2012*)

Working in a mass protest as a HCW is a unique experience, since a) politics is a dominant element in their nature, b) HCWs are targeted directly when they help protesters, and c) in some cases HCWs have not been there as a professional in the first place but as a protester; but after the attack of tear gas, plastic bullets and water-cannons they must suddenly change their societal role and act as professionals. Therefore being a HCW in a mass protest and the ethical problems related to that setting deserve to be explored in a separate study, possibly by using a phenomenological methodology. For that reason, 13 interviews with HCWs involved in the Gezi protests in June/July 2013 were extracted to be presented in a subsequent article.

The duration of healthcare provision during and after disasters was up to four years *(3 days to 4 years)*. The type of services provided were management of services, rapid health assessment, monitoring of post-acute phase, evaluation of the activities of the Red Crescent Society, search and rescue, distributing relief, establishing camps, environmental health, preventive care, curative care, psychological counseling, and training HCWs and the public.

The mean age of participants was 44.7 (24–64); mean experience in practice 20.7 years (up to 40 years), employed in institutions of schools of medicine, state hospitals, private hospitals, the National Medical Rescue Team, '112' ambulance services, and the municipality.

### Data collection and analysis

An initial semi-structured form developed by the researchers on the basis of literature and one pilot interview was carried out with the participation of a physician experienced as a disaster relief worker. Considering the delicate nature of the study topic, potential participants were first informed verbally regarding the aim and methods of the study and were allowed to ask questions before deciding whether to participate. They were also informed that the interview would be recorded but their identity and the institutions would be kept strictly confidential; that they could leave questions un-replied and quit the interview if they wish without giving a rationale. After that step, they were given a consent form explaining the aim and methods of the study along with the responsibilities of the researchers including confidentiality, and were allowed enough time to read it and ask questions again if there was any. Finally they were asked to sign the form if they decide to participate. We applied these steps to inform participants thoroughly and build a relationship based on trust, since we assumed that potential participants might refrain to reply some questions due to political nature of disasters.

All interviews were done individually by the researchers in the participants’ office for convenience; tape-recorded; and contemporaneous notes were taken when it was necessary. Three interviews were done by an internet-meeting program. Soon after the interviews, verbatim transcript were obtained and analyzed by the researchers, and used in the report after verification or correction of interviewees. The interviews lasted between 26 to 95 min.

Data collection and data analysis were done in an interrelated manner. Analysis of data was carried out concurrently with ongoing interviews in order to incorporate new insights into future interviews. Constant comparison of data was done and as the study proceeded, new questions relating to emerging themes were considered, and the interview form revised, i.e. the form was adapted to the type of disasters.

With the participants’ permission, the interviews were recorded and verbatim transcription done by a professional company. For thematic analysis, all transcripts were read and coded separately by all investigators. After initial reading, codes were classified as categories by comparing them across code lists. Codes were explored to see whether they were interrelated, and to reveal categories and concepts related to the ethical problems in disasters. Violations of rights and professional duties along with any ethical dilemmas were defined as ‘ethical problems’. Taking the ‘ethical problems’ as the core category, a selective coding process was carried out in order to consolidate the common themes and to depict an explanatory model showing the connection of the problems to their determinants related to disaster context. The Research Ethics Board of Uludag University School of Medicine approved the study (Jan 15, 2013–1/12), including the pilot interview.

### Limitations

One of the limitations of this study derived from the wide range of disasters that it aimed to cover. Different type of disasters can pose different value problems in diverse environments. In that sense, the coverage of this study might be questionable. For example, researchers failed to contact or persuade physicians working in Antakya, where the wounded fighters are brought for treatment from conflict zones in Syria; only two physicians accepted to participate. Another limitation was related to the defensive approach that members of the National Medical Rescue Team usually had; they were reluctant to take a critical position possibly due to being employees of Ministry of Health. On the other hand participants who were members of the disaster response teams of the Turkish Medical Association (TMA), which is dissenting of actual health policies as the main professional organization of physicians in the country, were eager to participate and might have been overly critical.

## Results

During and after the analysis, it was found out that the results of the study could be classified and presented under different categorizations according to disaster types (some have unique problems comparing to others), to the different parties of the problems, or to the periods of disasters (before-during-after). In order to see the connections between the problems and their determinants, we have considered the ethical problems on different levels or layers. Therefore we have classified the ethical problems that HCWs face, not just specific to healthcare provision, but also to the issues on a larger scale.

### Problems related to context

The participants mentioned various contextual or macro level problems. Those were related to public authorities’ paternalistic and defensive attitudes, media mindsets, relief organizations’ approaches, and insufficient guidelines and regulations which were defined as the traits of the context in which HCWs work.

#### Public authorities’ attitude

The reaction of public authorities during or after disaster is: *“everything is under control*, *all of the necessary actions will be taken by us as soon as possible”* [P1, Iz_A]. Along with a defensive attitude, progress is prevented by a resistance to cooperation with *“outsiders”*. This is especially distinct in large-scale disasters where international relief organizations come to the scene. Participants state that, primarily due to trust issues, public authorities usually do not like to share information nor authorize operations where they think it is inconvenient, such as refugee camps. In addition to the previous concern is the attitude of authorities to facilitate hiding sensitive information from the public, such as outbreaks of mumps or cholera: *“For example there was a mumps outbreak after the earthquake in one city but we recorded the cases as unrelated infections”*. (P2, Iz_C)

#### Media mindset

The mass media’s mindset and attitude after a disaster has been one of the most irritating, as charged by study participants, stating that reporters can recklessly block the relief efforts just to get closer images. Images of dead and wounded people might be published without any respect to personhood rights. The internet complicates and exacerbates this problem; their relatives and acquaintances may come across to those images even after many years: “*Everything on the internet stays there eternally*.” (P3, Izm_E). The mass media sometimes spreads unverified information and rumors, as evidenced by a participant who states: “*Just by seeing search-and-rescue teams using masks*, *they publish that there is an epidemic going on which is hidden from the public*.*”* (P4, Iz_B) Under political pressure, they may hide the truth intentionally and even spread misleading news such as *“Alawite doctors behave badly towards Sunni fighters and amputate them unnecessarily*.*”* (P5, Ant_A) Some participants emphasize that in order to transform the increased sentimentality of the general public into rating/sales, or to increase the relief to be sent, the media sometimes exaggerates a situation.

#### Relief organizations’ approach

Several participants complain that international relief organizations might approach in a paternalistic and imperious manner to local organizations, HCWs, and society: “*It is like ‘we know how to do it*, *you should provide us this and that*, *and this is all you need to do*.*’”* (P6, Ist_A) On the other hand, participants note a contradiction as they think that relief workers’ qualifications and medical interventions might be questionable, and some organizations do not arrive to the scene self-sufficient and therefore create an extra burden on the already-limited local resources. In addition to improper intervention, even quackery is a possibility: *“Once we were called by health authorities to evaluate the work of an international organization that cures stress disorder by touching the patient*. *After we examined their brochures etc*, *we realized that they were missionaries of Scientology*.*”* (P6, Iz_H) Another point was that they might act inattentively for referrals of patients to other provinces or abroad, neither sharing information nor writing epicrisis.

One of the things frequently mentioned by participants was that organizations use relief activities as a means for advancing their own agendas. These might be religious motivations, political aims such (as spying or gaining credibility and supporters), or raising funds: *“I saw that famous international aid organizations were not there only to help*. *Healthcare was the mask of the real business*. *There were other political or financial agendas; for example there was an auction for building a local airport*, *(and the focus was) whether that famous organization was going to win it or the other big one*.*”* (P6, Ist_A)

#### Ineffectiveness of humanitarian relief

The other theme that emerged during the interviews which could be classified under ‘unjust resource allocation’ was the ineffectiveness of humanitarian relief. The participants defined several problems related to sending, storing, and distributing relief. *“There was even offending aid*, *such as red lace g-strings*. *How can you expect earthquake victims to use those things*? *People sometimes send aid rather trying to ease their conscience*, *without thinking what a disaster victim needs*. *I’ve thought this was inhuman”*. (P7, Ank_B) Identification and classification of unsorted, not-universally labeled relief “*is a disaster on its own*” (P7, Ank_B), wasting labor and time, and causing pollution as well. *“Soon after an earthquake all day long I tried to classify the drug aid sent*, *thinking that it was better not to send those half used drugs from their drug-cupboard at home*. *Or*, *many drugs with non-Latin alphabet labels came… Uncategorized aid rather becomes a burden on aid workers*.*”* (P7, Ank B) Not registering the aid opens the ways to abuse and black marketing. Relief is usually not distributed by need, but just given away to whoever comes and demands it. Distributions are just “*thrown out from the trucks to the people*” in a humiliating way. “*For example I remember that one day on a muddy street*, *people were running after a truck in order to get the aid*. *Some of the aid in the truck was vital such as tents or heating devices*, *etc*.” (P8, Iz_D). The participants also mentioned that politicians abuse their power for the sake of their own agenda, which causes discrimination in distributing relief on the basis of religion, ethnicity, political views, etc. Discrimination involves HCWs and the facilities to be provided to victims.

#### Insufficient guidelines and regulations

The majority of the participants think that professional guidelines and regulations do not provide enough guidance in disaster settings. Foreign organizations usually implement their own codes, which might be contradictory to local values and conditions. “*On the other hand*, *international aid organizations should learn the cultural features of the affected country better before going there*.” (P9, Iz_G) They also think that existing guidelines are not taught well, and even though they are known it is not easy to follow them due to the circumstances and pressures in disaster settings. “*Because conditions are very different from a routine situation*. *You are in the middle of chaos*. *Sometimes you can experience difficulty in following the rules*.” (P10, Bu_C) Several suggestions were made by the participants for improving the guidelines, which also show the problematic areas of guidelines and regulations:

- Regulations should consider that disasters go beyond “9 to 5”; they can cover 24 hours. They also should comprise a wide range of relationships.
- Need should be the basis for organization and provision of healthcare.
- HCWs should be trained and prepared for disasters and behaviors of affected people.
- There is a need for big umbrella-like international regulation, which defines minimum standards for all parties including;
  ∘ Duty to prioritize victims’ interests
  ∘ Equal distribution of relief
  ∘ Avoidance of discrimination
  ∘ The limits of each party and the need for cooperation
  ∘ Creating suitable settings for providing service and professional autonomy
  ∘ Providing care to disabled and other vulnerable groups
  ∘ The responsibilities of expatriate relief organizations and workers
  ∘ Legal dimensions, especially regarding malpractice

#### Mismanagement due to unpreparedness

The participants repeatedly state that disorganization and problems of management complicate the situation in disasters where access to services is already diminished in addition to the preexisting availability and accessibility problems. *“If you talk about malpractice of a physician who intubated the patient*, *then you should also talk about malpractice of the mayor who gave the approval for the hospital building severely damaged in an earthquake*. *Eventually*, *the governor of the province who did not put initiatives in preparing provincial disaster plans contributes to the chaotic situation*.*”* (P7, Ank_B) One participant emphasized that implementing ethical principles is strictly linked to disaster management: *“Incorporating ethical principles into practice basically depends on the effectiveness of humanitarian aid management”* (P8, Ank_C)

One of the main reasons for mismanagement is stated as the lack of rapid health assessment to determine actual needs, which can cause misallocation of resources while excessive and useless resources pile up in one place, but no care in another, eventually leading to preventable suffering and death. “*Mostly curative services are prioritized in disasters*. *Generally SAR teams are sent and they begin to rescue*. *But the most critical thing is making a preliminary evaluation*, *and then deciding on allocating resources based on the evaluation data*.*”* (P11, Ad_A).

In addition to lack of rapid health assessment, the participants emphasize that the other dimension of unpreparedness is the lack of disaster-specific organization. Therefore, when a disaster strikes HCWs and resources are sent to the area *“without thinking”* which contributes to chaotic conditions. *“This impulsive reaction limits healthcare mainly to curative services*, *and causes negligence of vulnerable groups such as children*, *disabled people and refugees*.*”* (P3, Iz_E) When there is no advance planning, damaged healthcare institutions continue to be used, saved victims are referred to random hospitals or even other cities, healthcare-tents in camps are not suitable for service nor safe.

A sudden appointment of workers from various cities without any personal preparation decreases their motivation and obstructs teamwork. Unplanned employment of workers without a work description causes waste of workforce and feelings of futility and anger: *“People stray around like drifting mines”* (P1, Iz_A) They start questioning, asking themselves *“Am I really needed here*, *touching people’s lives meaningfully*, *or am I just wasting resources*?*”* (P11, Ad_A) Unjustifiable usage of resources was defined as a source of major ethical tension. The participants also stated that voluntarism itself might be a source of problems, motivation from heroic feelings and altruism might lead to higher risk-taking behavior and therefore malpractice. “*You take the road to a disaster area with a serious adrenalin load*. *You notice its negative effects only after the 3rd day*. *I drove to an earthquake area which is a 20 hour car ride*, *and I remember I saw 170–180 km/hour speeds on those curved roads*.” (P12, Bu_B) They connect it to another problem as well, wherein HCWs are aware of their need to take a break, while on the other hand resting may be regarded as unethical. *“Although we sometimes thought of having some rest*, *but we did not*, *saying that there are wounded people*, *how could you stop caring for them*? *This threatens both your and people’s health*. *Any mistake is still malpractice even if you are tired*.*”* (P4, Iz_B)

All of those dimensions of unpreparedness mentioned by the participants lead to mismanagement, which is closely linked to unjust resource allocation and diminished access to needed services. In some such cases, disaster victims can even be harmed by relief activities.

### Problems in patient-HCW relationship

The participants have told us that they encountered ethical problems related to patient-HCW relationships, or micro level issues, such as professional incompetence, difficulties of determining the limits of duty to care, obtaining informed consent, and violations of confidentiality and privacy.

#### Professional competence

The participants report concern of medical competence as a potential source of ethical problems. They state that in general HCWs sent to disaster settings are not properly trained specifically for disasters. Half of the participants state that they have had limited disaster medicine training mainly through short theoretical lectures during their undergraduate education. Some of them state that lack of proper education makes it difficult to be benevolent to victims: “*In any case*, *there is no such education routinely provided in medical schools in Turkey*.” (P5, Ant_A) Therefore some conclude that improving medical education for training HCWs specifically for disaster conditions is “*a moral duty*. *Especially for the clinical practitioners*, *learning of abstract ethics concepts should be supported with real life cases and applications*.*”* (P9, Iz_G)

However, regarding legal liability that might emerge due to medical intervention in disasters, a majority of the participants think that they should not be held liable while they work to save lives, mainly because a) any available tools for intervention are very limited, so an altered standard of care, even expanding the scope of practice is justifiable, b) they are a patient’s “*best shot*” under those conditions, c) their specialty might not cover emergency treatment and/or they may have lost necessary knowledge and skills for first aid over the years. So not intervening is equal to removing the only chance a patient has, no matter how slight it is, and since their intention is helping in good will, it would be unfair to hold them liable for malpractice. On the other hand, a few participants state that some limits should be defined by using certain measures, “*otherwise HCWs might act recklessly*”. (P9, Ank_D) For example, first aid should be provided properly in all circumstances and therefore HCWs should be held fully responsible for the harm they may cause, but there might be exceptions in the case of emergency treatment. “*You as a physician have the instinct of helping people*, *which might sometimes mean going beyond your professional limits*. *Especially during the very acute phase of disasters*, *when there is no other healthcare worker*, *whatever your specialty is*, *we have the responsibility to care*. *Under these conditions*, *a physician trying to do their best might face exceptions*. *However in any case physicians generally should be responsible for their actions*.” (P11, Ad_A)

One-third of the participants state that they received some educational lectures on medical ethics during undergraduate education, while none of them were directly related to disaster bioethics. However they do not usually consider this an important problem, and it is thought that HCWs should be allowed more flexible action with regards to patient rights and professional ethics codes during disasters, in order to be able to perform life-saving activities for people's vital needs. Especially in the acute phase when saving lives is the priority, “*ethics is not thought of*”. (P18, Bu_A) Another justification is that they generally think that professional morality rules that they have already internalized and become accustomed to in their daily routine would provide appropriate guidance. *“Approaching from a humanistic perspective”*, *“always having good intentions”*, *“protecting the right to live”*, *“knowing the necessity for giving the priority to a patient’s best interests*” are considered as “*instructive enough*” regarding ethical decision-making during disasters. They feel that the ethical aspects of professional practice become more prominent after the acute phase and a more analytical approach can be adopted.

#### Triage

Some of the participants report that triage is not applied properly or not applied at all, which could be harmful or even cause preventable deaths: “*I never come across triage areas in any of the disasters I was involved in*. *(…) while the hospital garden was full of crowds*, *even aside from triage*, *nobody even knew who did what*. *There was neither a triage area nor a sense of triage*.” (P14, Ank_C) A physician mentioned that prioritizing children over adults for rescuing under the rubble after an earthquake is a controversial decision. Another participant defined treating patients with a ‘black code’ as futile, since it is fully based on personal conscience not medical necessity, wastes limited resources and is therefore ethically problematic.

#### Determining the limits of duty to care

Under tragic conditions, determining the limits of duty to care while protecting a patient’s best interest and the duty of non-discrimination might create severe moral tension. One of the conditions that might shake the grounds of duty to care is defined as the case of insufficiency of known protective measures and treatment in recently emerging, highly fatal communicable diseases, as in a SARS outbreak. One participant felt that there is no duty to care in that case, and suggested employing only volunteers to serve those patients, while another suggested volunteer or not, physicians should be assigned by the state if the right to health is to be protected. The same physician, who had developed Crimean-Congo hemorrhagic fever–a highly fatal and non-treatable communicable disease when trying to save his patient’s life also stated that there is no limit for duty to care in this case and put forward the following justifications for his thoughts: “*I would do it again whenever it is needed*. *I cannot look at myself in the mirror if I don’t help someone that could be rescued with my intervention*. *It would destroy my honor*. *Then*, *there would be no meaning to living*… *Patients have no other place to go*. *We voluntarily put our hands under this stone* (by choosing the profession and continuing to practice)”. (P15, Ank_F)

The conflict of a physician’s personal values and emotions with a patient’s personality and actions might be another reason to refuse care. One of the participants who worked in a hospital serving jihadists warring in Syria related: “*Sunni jihadists who were killing*, *raping and decapitating Alawites came to hospitals in Turkey*, *refused to be treated by Alawite physicians*, *insulted them*, *and even sexually harassed nurses*. *Usually we can’t think about whether or not he will continue to kill or rape once he gets healed; we do what we need to do to serve because he is a human being*. *As far as I heard only one physician refused to provide care for Sunni jihadists because of those insults*”. (P5, Ant_A) Also some foreigners, “*supposedly physicians from Syria*”, come to hospitals and accuse HCWs of discriminating against jihadists. They even intrude into operating theaters and threaten HCWs stating “*refer the patient this or that center*, *or you will pay for it*”. (P5, Ant_A) On the other hand, authorities put pressure with prejudices on doctors' professional autonomy. “*The director of my hospital gathered all our surgeons and warned us not to amputate jihadists from neighboring countries unnecessarily*, *and to behave nicely toward them*, *otherwise we would be tried in war courts*.” (P5, Ant_A)

The general panic during an emergency might also influence HCWs and cause them to ignore a patients’ life and security: *“During the TUPRAS* (a huge petroleum plant) *fire*, *after the news about evacuation of nearby districts*, *personnel left the hospital immediately*, *while leaving some ICU patients as they were*.*”* (P14, AnkC)

#### Respecting patient’s autonomy

The participants usually defend a paternalistic approach based on the extraordinary conditions of disasters: *“This is the common thinking*: *people exposed to disasters cannot think rationally*, *I am there to help them and they know this already*, *therefore I can and I should decide on behalf of them*.” (P4, Iz_B) In order to ease their workload, they claim that informed consent should not be defined as a professional duty in the first three to five days since it is the “*initial chaotic phase*” with very limited resources, and the affected people are often in an *“unusual”* psychological state. However, a few participants expressed that intervention with limited and primitive resources sometimes requires one to obtain consent. The provision of healthcare in tent-cities, which is similar to routine polyclinic service, is also seen as a circumstance that requires informed consent.

Some of the participants state that obtaining informed consent might be too difficult when the patient is a refugee and doesn’t know the local language. They relate that even the consent forms prepared in their own languages are not enough to make an informed choice, since refugees are desperately in need and sign anything they are required to. *“In the beginning there were no Arabic interpreters to explain the procedure*, *vital risks and consequences such as amputation of the leg*, *and we had severe difficulties*. *Now some Arabic speaking staff of the hospital were recruited as interpreters and consent forms were translated*, *so we could at least establish communication*. *But even the interpreters and consent forms are sometimes not helpful enough*. *If you ask me if the availability of interpreters helps people to fully understand or not*, *I’d say there is no other choice*. *Whether the patients understand or not*, *they reluctantly accept whatever we propose*. *On the other hand*, *for us there is no other place to transfer the patient and we try to complete the treatment successfully*.*”* (P5, Ant_A)

#### Violations of confidentiality and privacy

Keeping patient information safe is generally neglected according to the participants. Patient files are not kept in lockers, and this creates a risk especially for domestic violence victims and drug-addicts. “*One can easily access the files if they’d like to*”. (P17, Iz_H) Securing the confidentiality of data after leaving the disaster area is even more problematic. “*Those patient records were not kept in locked cupboards or rooms*. *We established a very detailed registry system including patients’ open names*, *number of tent*, *and existence of any drug addiction or domestic violence*. *I still don’t know what happened to those records*.*”* (P17, Iz_H).

The participants state that the proximity of tents, separating service areas only with screens, and the sudden entrance of people into healthcare tents violates patient privacy. Using interpreters sometimes prevents patients from expressing themselves fully, especially when there is no woman interpreter available. Also, having interpreters in examination rooms damages privacy. The extensive use of smartphones by HCWs also violates privacy when they recklessly share affected and wounded people’s images on social media after a disaster.

## Discussion

In this study, the aim is to understand the types, nature and determinants of ethical problems emerging in disaster settings; therefore we have limited interpretation of the results within this viewpoint. However, we realize that there are many questions to be answered relating to ethical problems and the arguments of participants, so we have stated them as the first steps for an ethical analysis of the dilemmas.

### On a micro-level

With regards to the patient-HCW relationship level, or ‘micro-level’, a lack of medical education specific to disasters emerges as one of the most important sources of problems. The participants were partly aware of this unmet need for education. This is particularly true for triage, possibly due to limited/non-present knowledge or practice in it, despite the fact that triage is one of the most prominent topics in disaster bioethics. Similarly, in a study carried out with 205 physicians in Turkey, only 36.3% of participants stated that they had applied triage principles [15]. In fact, lack of specific education on disaster medicine is a widespread problem all over the world [16–18]. Today everyone is aware of the increasing risk of major accidents and disasters, and the importance of active preparation–including training; however, nobody invests in it [19]. Not having the appropriate knowledge and skills causes preventable deaths and injuries, therefore it is possible to claim that training HCWs on disaster medicine is a must.

- Is providing specific training of disaster medicine to HCWs to be sent to disaster settings a moral duty?
- If so, who are the parties that are responsible for this duty?
- If so, is sending uneducated HCWs to disaster settings immoral?
- When triaging patients, is favoritism towards younger victims morally justifiable in some cases? Or is it age-discrimination regardless of conditions?
- Should ‘easing personal consciousness’ be a justification for futile treatment?

Ironic as it might seem, while the participants state that lack of education is an important problem because it might cause preventable harm, a majority of them tell us that they should not be held liable for malpractice. Their argument is based on four premises; that they are the “best shot” of the patient; resources are very limited; they might not be properly educated for that specific and urgent intervention; and they are helping with good intention. As a matter of fact, this is still a controversial issue in medical ethics and law disciplines [20–22]. Analyzing all of these arguments, including the opposing ones claiming that HCWs should be liable to a certain degree, might contribute to the current debate.

- Should a HCW be held legally liable for any harm caused by emergency treatment in disaster settings, when;
  ∘ the available resources are not appropriate for the necessary intervention,
  ∘ the HCW is not properly trained for that intervention, while he/she is the only one available to perform the intervention.
  ∘ the intention of the HCW is purely altruistic.
- If there should be a line to hold HCW liable, then how and where should be that line to be drawn? Should the difference between ‘first aid’ and ‘emergency treatment’ be a measure?
- How should we determine the liability due to healthcare-related harm? (i.e., because of the lack of triage system)

Contrary to their opinion on medical competence, the participants believe that lack of education on medical ethics is not an issue creating significant harm. They usually shift to a utilitarian approach regardless of their prior understanding of what constitutes ‘good’, asserting that saving a maximum number of lives possible has priority over all other concerns. Based on this approach, they believe that they should not be forced to lose precious time by intervening with binding ethical codes, rules or principles. This is also true for their mindset about the duty to obtain informed consent, which seems like one of the least important amongst professional obligations, especially if the situation requires urgent intervention. A study exploring the question of whether the autonomy principle is taken into account or not found a similar rate (4.4%) [15]. Participants think that they should not be obliged to obtain consent for up to five days in the aftermath of a disaster, because ‘beneficence’ is above ‘autonomy’ in those circumstances. This approach is worth further detailed evaluation, even though it might seem obvious, since a) it ignores ‘autonomy’ for the sake of public good which might be linked to some kind of a survival instinct, while claiming that it is the most beneficent thing to do for that particular person as well, b) this approach may be called *communitarian*, which asserts that the good of the individual is inseparably linked to the good of the community, rather than *utilitarian*, and c) if *communitarianism* should be the prevailing approach in disaster medicine instead of *utilitarianism*, then its reflections and effects should be explored both theoretically and practically. Furthermore, the majority think that the professional values and decision-making capacity they had already acquired in daily practice should be enough for extraordinary situations as well, along with having a “good intention” as a “helper”. Similar to their opinion on malpractice liability, as long as their intention is altruistic, then there is no way for doing ‘evil’, therefore they should be able to do whatever they think benevolent for the victim. This approach seems eclectic because it adopts a Kantian approach by defining ‘good’ with a categorical imperative of having a good will, while it overrides autonomy, which is also based on the Kantian approach in a paternalistic way. Another argument for overriding the victim’s autonomy is based on the presumption that disaster victims lose their decision-making capacity temporarily because of the shock they have experienced. Although it might be true for the majority, whether it is true for every single individual is a question to be explored scientifically. In summary, considering that the special circumstances of disaster settings augment the unequal nature of the relationship between the caregiver who has the specific resources and the patient who needs those resources more than ever, the duty to obtain informed consent is one of the topics that should be investigated thoroughly.

- Does helping / having good intentions, or coming from outside as a “helper” provide a positional superiority for moral aspects?
- Should a paternalistic approach be adopted in certain conditions? Is there a need for a more flexible approach to informed consent during the initial days after a disaster?
- Is there a consensus for using a utilitarian approach in public health emergencies?
- Should communitarianism be adopted instead of utilitarianism while working in disaster settings? What kind of professional duties would it impose different from utilitarianism?

Determining the limits of duty to care in outbreaks, armed conflicts and emergencies might create profound tension between a patient’s best interest and personal concerns/values. Recent cases of some HCWs who refused to treat Ebola patients, went on strike and left the country, and Iraqi physicians who refused to treat ISIS militants and were killed for that reason have brought questions regarding the duty to care into the agenda [23–25]. Some argue that physicians should not “pick” their patients. As William Boghurst (who did not flee when the Great Plague struck London in the 17th century) wrote, “Every man that undertakes to be of a profession or takes on himself an office must take all parts of it, the good and the evil, the pleasure and the pain, the profit and the inconveniences all together and not pick and choose; for Ministers must preach, Captains must fight and Physicians attend upon the sick” [26]. However, there are no clear guidelines on how to act in such dramatic circumstances, and there is an urgent need of a wide consensus on this unsettled issue, guiding HCWs on the line between duty and heroism, right to refuse to care—if there is any, and how those patients should be served. As another dimension of the issue, political pressure on HCWs’ professional autonomy should be taken into account.

- Under which conditions do the grounds of duty to care become controversial? Could we define a limit? Based on which criteria?
  ∘ Which attributes should a communicable disease be included or excluded? *(Availability of efficient prevention methods and/or treatment methods*, *morbidity and mortality rates*, *social stigma…)*
  ∘ What if there is violence towards HCWs?
  ∘ What if there is an immediate threat to HCWs?
  ∘ When do personal values conflict with professional values?
- How can the *right to health* be protected under those circumstances? Who should care for diseases outside the scope of duty to care?
- When their professional autonomy is violated by political pressure, how should HCWs determine the limits of duty to protect professional autonomy?

Breaches of confidentiality and privacy seem like another neglected problem. The nature of disaster conditions might prevent HCWs caring about the necessary conditions for respecting confidentiality and privacy. It should always be kept in mind however that disaster conditions are not limited to urgent treatment; they also cover provision of healthcare in temporary settlements like camps or field hospitals. Keeping patient information safe and respecting personal rights should be dealt with as an issue to be managed carefully, so that there should be regulations for even the usage of social media. Ethics teaching should aim to contribute to the ethical sensitivity of HCWs regarding this issue. Providing professional and educated interpreters is another requirement for a service respectful to patient rights.

- Who are the responsible parties to keep patient data safe and secure? Who should be able to access to which kind of data and when?
- What are the responsibilities of humanitarian organizations and authorities regarding data sharing?
- How should the data be handled after the relief operation is over in the field?
- What are the conditions under which we can breach confidentiality in disaster settings? Are they different than normal healthcare provision?

### Problems on macro level

As well as the problems on the micro level, the participants mention factors on the macro level that surround the patient-HCW relationship: public authorities’ and politicians’ attitudes, mismanagement of services, problems related to relief, relief organizations, guidelines, and media. In fact, this is the central theme of the participants’ narratives, when asked to relate the ethical challenges they face in disaster settings.

Disaster conditions inevitably create gaps in the hegemonic area of the power of authorities, who attempt to restore command immediately and decisively. Therefore public authorities take a ‘paternalistic’ position, claiming everything is under their control as if omnipotent. This can seem partly justifiable in order to prevent unfounded panic among the public, but because of unwillingness for cooperation and criticism, opportunities to improve services are missed, and at least some of the problems are ignored. Along with a defensive attitude which can lead the authorities to hide information, their approach might jeopardize public health and decrease their own credibility which contributes to any actual chaos. As much as possible, transparency and accountability seem like vital conditions for community participation and mitigation. Also, politicians might abuse their power for the sake of their own political or individual agendas. Although this seems unpreventable to a degree, violations on basic rights such as discrimination and pressures on HCWs such as threatening cannot be justified in any case.

More than 90% of disasters occur in developing and underdeveloped countries, where inequities are profound and resources are already limited or simply nonexistent [27, 28]. As O’Mathuna put it, “Addressing such inequalities in disaster risk reduction is an ethical issue. Disaster preparedness must therefore go deeper than preparing to rescue people after the disaster strikes. It must go to the core of why such social inequalities exist and are permitted to continue” [29]. Therefore, in addition to those negative factors mentioned above, people who hold authority and power in their hands for the public good have positive responsibilities as well, such as providing proper housing, decreasing inequality, and implementing policies for preparedness to prevent harm as much as possible, both before and after disasters.

- Under which conditions and to what degree is it justifiable, if ever, for public authorities to:
  ∘ act as if everything is under their control, so they don’t need any help from outside, and
  ∘ hide information from the public?
- Which duties do HCWs have to protect public health? Do they have a duty to inform society?
- What should HCWs do if they are requested to hide information related to public health?
- For the harm related disasters, what are the ethical responsibilities of people with authority and power to make policies and decisions? How can it be measured?

Not being prepared for providing healthcare services when a disaster strike leads to mismanagement of relief activities. Hunt showed that organizational forms and structures in disasters shape everyday moral experience [30]. Sending HCWs and relief without doing a rapid health assessment or considering actual needs, along with the lack of disaster-specific organization of services linked to the misallocation of resources and therefore diminished access to care, and HCWs become a part of the chaos. Secondly, HCWs should be trained properly as mentioned before, and be sent contingent upon need-based planning. Otherwise the attitude arises that they have wasted already-limited resources without any meaningful contribution, and their psychological mood can finally become one of anger and exhaustion. Also, an HCW’s willingness while performing their task in a disaster zone is another point to be considered carefully. In a study, healthcare workers’ willingness to work during a disaster is found to vary with the type of event, ranging from a high of 84% during a mass casualty incident to a low of 48% during a SARS outbreak [31]. On the other hand, motivations behind volunteering in rescue or relief activities might not always be justifiable. They may be personal, such as a ‘life of adventure’, universal and ethical, such as ‘desire to participate in meaningful change’ or ‘promotion of human rights’, or religious or accidental [32]. The ‘heroism’ instinct could be another factor. For those in search of adventure or worse disaster tourism, it provides ‘adrenalin of war without being at war’ [32].

In sum, the finding of this study suggests that relief healthcare service should be prepared beforehand in all its dimensions in order to serve effectively and justly in disasters.

- Should HCWs be assigned if they did not volunteer? Under which conditions?
- What underlying motivations for voluntarism can be justified?
- How should a service be organized to be sensitive to the needs of vulnerable populations?
- Should HCWs be allowed to work in acute phases if personally affected by that disaster?

Sufficiency of guidance for decision-making when facing an ethical problem was another issue indicated by the participants. There are several guidelines specific to disaster settings such as World Medical Association Statement on Medical Ethics in the Event of Disasters, the Code of Conduct for the International Red Cross and Red Crescent Movement (ICRC), the Sphere Project’s Humanitarian Charter and Minimum Standards in Humanitarian Response, MSF’s Charter and Principles, or Ethical Principles on Disaster Risk Reduction and People's Resilience of Council of Europe [33–37]. However, it is argued that guidelines are “not known, not universal, not applicable in early response phase, providing conflicting moral injunctions, and not decreasing moral stress” [38]. There is also a need for a consensus on a virtue-based, yet practical ethical approach to medical care under such extreme conditions, as Holt suggested [39]. There are some suggestions in the literature formulated for decision-making [40–42]. In addition, Lepora suggests usage of checklists for HCWs as a practical tool for “what to do when, what to discuss, what not to do” [43]. Yet their effectiveness is to be researched [10].

Another common problem with the guidelines is that they usually focus on micro-level resource allocation problems. As the participants of this study suggest, the guidelines should be improved in a way to cover all issues other than clinical ones, including the responsibilities of different parties. A group of experts who had explored the Code of Conduct of ICRC recommended a similar approach to improve it, for instance [44]:

- Updated definition of disaster
- The code should strongly promote disaster risk reduction, capacity building, participation of local communities, and respect for local culture
- Gender issues need central prominence
- The code should mention the importance of humanitarian needs assessment
- More emphasis is needed on vulnerable and marginalized groups in different cultural settings
- Disaster relief in conflicts needs elaboration
- Prevention and reduction of risk need to be addressed
- Ethical issues for research in disaster settings and with humanitarian aid need to be addressed
- The code promotes accountability to donors and recipients but needs more guidance to deal with unethical practices such as discrimination, favoritism or corruption, and various conflicts of interest
- Issues related to liability of aid providers and responders need to be included.
- Importance of cooperation with local governments is highlighted but local organizations should also be mentioned

On the other hand, it is a fact that written codes and regulations cannot cover every aspects of life nor are they as dynamic as life. If the guidelines and alike could provide enough guidance in all circumstances, then there would be no ethical dilemmas. They cannot be valid in every circumstance every time; there will always be a critique of insufficiency. Therefore a) “a code of ethics should serve only as a guide for ethical reasoning” [45], and b) virtue-based training on implementing guidelines and finding/creating the right action to protect the right to health and professional values is important.

The ineffectiveness of humanitarian relief is another topic as a source of ethical problems revealed with this study that leads to unjust resource allocation and diminished access to care. Sending unsorted relief instantly without basing distribution on needs assessment wastes time and effort and causes environmental pollution. The reasons behind sending relief without thinking might be various, from altruism to “*easing one’s conscience*”; the line between them might not be clear as well. Many of the observable facts about giving/donating could not be explained by pure altruism [46]. It could be “herding behavior” [47], or giving charity or sending relief during humanitarian crises can serve as a conscience pacifier at a micro level; “I have done my bit for mankind so I don’t need to bother with structural inequalities” [32]. In any case, over the years this has been a prevailing, well-known problem. “Humanitarian assistance must be needs-driven, not resource-driven,” as it was put in the training material of the UNDP [48]. Distributing relief might be even more important, since its mismanagement can cause the humiliation of victims, black-markets, and a bias in favor of healthy and powerful persons instead of more needy. “In all disaster situations, people who are more powerful before the disaster will be able to use relief aid to increase their power relative to those who were weak” [48]. Pressure from politicians for distributing relief to their voters is a clear case of discrimination, and violation of the right to health.

- Does altruism include a sort of egotism and self-interest? If so, does it make a difference in practice regarding relief and aid activities in general?
- Do HCWs have any responsibility in the just distribution of relief? If so, what are the limits? (Assessment of needs, informing authorities about these needs, etc)
- Are healthcare and HCWs just ‘instruments’ or a ‘relief item’ to be used by governmental authorities as they see fit? How is the medical profession different from a blanket or a tent regarding fulfillment of function?
- How should HCWs respond when they are pressed to be a tool of political discrimination?

Non-discrimination is a guiding principle also for relief organizations. According to the Code of Conduct of the ICRC, “Aid is given regardless of race, creed or nationality of the recipients and without adverse distinction of any kind. Aid priorities are calculated on the basis of need alone. Aid will not be used to further a particular political or religious standpoint.” [34] Although humanitarian aid should not be used as an instrument of religious, political or financial motivations, the research participants gave many counter examples in practice, in which relief organizations discriminate according to their own priorities. Lyon defines this phenomenon saying “altruistic interventions are often blurred with self-interested power pursuits” [49]. Likewise, Stehrenberger and Goltermann emphasize that disaster medicine “shifted to foreign operations often in the global south conducted by Western agencies during disasters after 1945 and increasingly after the end of the Cold War.” [50]. They state that it is very important to understand “the historicity of disaster medicine as a political phenomenon and of the discourses denying its political nature”. Thus, the instrumentalization of humanitarian activities, together with the question of whether humanitarian aid itself is an instrument or not seem like significant points for moral deliberation. It is possible to argue that the instrumentalization of relief activities for the organizations’ own agendas devalues the concepts of ‘helping’ and ‘solidarity’, and decreases trust toward relief organizations and other societies. Paternalistic and non-cooperative attitudes of international relief organizations might also be related to this issue. Ironically, their preparedness and competence might be so questionable as to even possibly see examples of quackery.

- What are the responsibilities and duties of humanitarian organizations towards disaster victims, local aid workers, authorities, and other NGOs? Is it possible to develop common standards of conduct? Is it possible to implement them effectively?
- Could instrumentalization of humanitarian aid be justified? Is it realistic to expect relief organizations not to pursue their own agenda? To what extent is it acceptable? Which motives are acceptable?

Another macro-level factor is the mindset of the media, when it prioritizes its own interests over public good. While media has the vital function of informing people and broadcasting the problems in the aftermath of disasters, they might not respect their own ethical codes. They can publish unverified information, exaggerate situations, and violate personhood rights to increase ratings. Therefore HCWs try to stay distant and protect patients from media if the right to be informed properly and timely is being violated and the reliability of any information diminishes. The mainstream media’s relationship with political power is also problematic when it comes to keeping independence and integrity intact. In this study there have even been examples of hostile feelings being spread among people by publishing false accusations toward physicians. It is possible to remind journalists of their responsibilities to the public and ethical codes at this point; but at the same time how naive or realistic it would be is an issue to consider. Also, as social media thrives in the digital age, whether ‘citizen journalism’ could provide a meaningful contribution towards the right to know, and whether it could shake up the status quo of the media seem like questions worth deep thought.

- Which information about disasters should be received from the media? What information is newsworthy?
- How can we define the limits of the ‘right to share the information’ in disaster settings? Which images and information can be used without consent?
- What are the responsibilities of different parties, including HCWs, to protect the dignity of victims from the media?
- What should be done for an independent medium that provides news about facts in the aftermath of a disaster?

### An explanatory model

**Fig 1:**
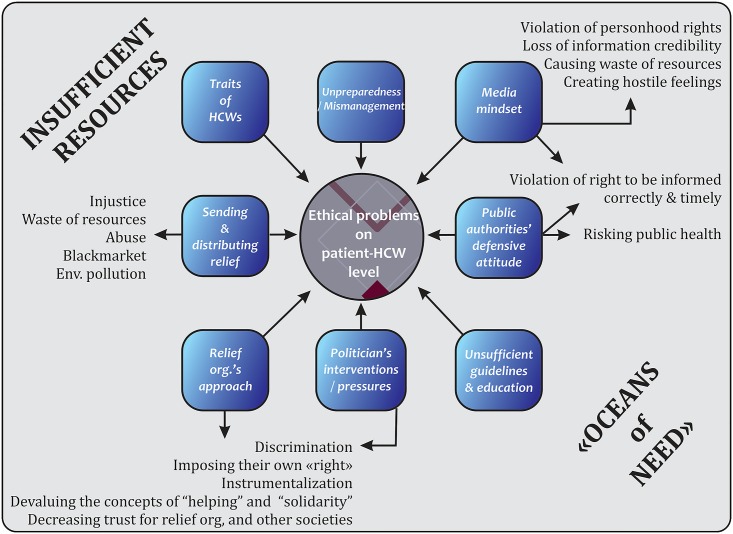
An explanatory model for ethical problems in disaster settings*. * The term “Oceans of need” was quoted by Hunt [3] from M. Michael & A.B. Zwi [51].

The findings of this study have provided insights on the variety and nature of ethical problems in disaster settings. They can be summarized with an explanatory model including the context, the features of moral agents, and the problems emerging at the level of the patient-HCW relationship (Fig 1).

When initially considering ethical problems in disaster settings one might tend to isolate them into issues of life & death during the acute phase. However, our study reveals that ethical problems are not limited to triage level decisions amid tragic scenes, nor to a few days. Disaster conditions generate various ethical challenges on a variety of levels including the macro level which can emerge or persist into the post-acute phase, years in some instance. Secondly, elements on the macro level create ethical problems on a larger scale, including factors such as discrimination, violation of personhood rights and waste of limited resources. And thirdly, it is possible to argue that these factors on the macro level are also a directly cause of, or facilitator of, the emergence of ethical problems on the micro-level. This is especially true when HCWs know what course of action is ethically justifiable, but are unable to act or hesitate because of the factors surrounding the micro-level.

Considering the model, it is possible to infer two conclusions. First, if the factors on the macro level affect the ethical problems on both levels, then intervening in those factors would have a significant effect on problem prevention. Preparedness especially seems as an important factor for avoiding moral tension and violations. Geale expressed this necessity, stating “the most ethical approach would be to spend time and money in mitigation of disasters through prevention of, and planning for those disasters that are unavoidable” [52]. Therefore, it could be argued that policy-makers and healthcare authorities have a moral responsibility in this regard. Improving guidelines and professional codes, and educating HCWs both for medical and ethical competence are also areas with potential for significant improvement. Training on how to manage crisis situations would be helpful for ethically justifiable decision-making and to increase benevolence.

Another point to emphasize is that the nature of ethical problems emerging in disaster settings have a political dimension. The political characteristics and power relationships of the society that disaster strikes play an important role on macro level factors such as public authorities’ attitude, politicians’ interventions and media’s approach. Besides, when a disaster hits, the hierarchic balance and hegemony networks inherent in that society are disturbed, and the interests of different parties conflict. “Politicians seek political support, caregiving institutions want additional resources, and various first-responder agencies will maneuver for authority and leadership” [53]. This is also true for the relationships between HCWs and the other actors of disaster scenes, since HCWs might be in a position to protect the patient’s best interest and professional duties against conflicting demands and pressures. In addition, the discourse and practice of humanitarianism has become increasingly politicized [54]. Recognizing that ethical problems in disaster settings have a political nature inevitably necessitates some professional responsibility of HCWs, such as rights advocacy, creating awareness, and informing authorities and the public about problems. It also requires Bioethics as a discipline to have a holistic view and to consider the relationship between the macro and micro levels in its ethical analysis and normative claims regarding disaster settings. Adopting a biopolitical approach would entail a role of providing guidance to HCWs, which includes not just a definition of an abstract *good*, but also advocating the right thing to do in the name of right to health and professional duties. Otherwise, mainstream debates of Bioethics will continue to limit the moral deliberations to the clinical setting. It is clear that ignoring the very nature of these ethical problems is contradictory to the principle of benevolence.

Further studies are needed to deepen the understanding of the role of macro factors on both levels. Studies and analysis should take into consideration the pre-disaster conditions, especially social injustice and inequality in order to improve the explanatory model. Besides, we did not focus on the analysis of the cases revealed in this study one by one, in order to consider how guidelines and professional codes are instructive, what options to act are open to HCWs in each specific case, and which option is relatively best and by which justification. These concerns are certainly necessary to be addressed after this descriptive study through further ethics inquiries. In addition, we think that the questions we have defined with this study should be dealt with through Bioethics, both for developing guidelines and education programs, and guiding HCWs in the field for ethically justifiable decision-making.
